# 基于点击化学的色谱分离材料研究新进展

**DOI:** 10.3724/SP.J.1123.2022.11015

**Published:** 2023-01-08

**Authors:** Jiabi XU, Yue CHENG, Xinling LU, Xiaoning JIN, Yong WANG

**Affiliations:** 天津大学理学院, 天津 300350; School of Science, Tianjin University, Tianjin 300350, China

**Keywords:** 点击化学, 色谱分离材料, 柱色谱, 膜色谱, click chemistry, chromatographic separation materials, column chromatography, membrane chromatography

## Abstract

自诺贝尔奖获得者Sharpless教授2001年首次提出点击化学概念以来,该类反应凭借条件温和、反应迅速、产量高、副产物少、分离提纯简单等优势,迅速拓展至材料和生命等诸多科学领域,成为一种强大的模块化合成工具。目前,点击化学反应已成为设计制备分离材料的重要手段,展现出蓬勃发展的现状。本文首先简要地回顾了点击化学的发展历程并介绍了其独特优势,然后聚焦于柱色谱和膜色谱两大分离领域,系统地综述了近5年发表的基于点击化学的色谱分离材料相关报道,重点归纳了叠氮-炔、巯基-烯和巯基-炔这3种常见点击反应类型在色谱分离材料研究中的最新进展,最后对点击化学在开发高效分离材料方面的发展前景进行了展望。

## Abstract (en)

Since Nobel Laureate K. B. Sharpless first introduced the concept of click chemistry in 2001, such reactions have become a powerful modular synthesis tool. Click chemistry reactions have rapidly expanded into many scientific fields, such as materials and life science, owing to their distinct advantages, which include mild conditions, fast reaction rates, high yields, low by-product generation, and simple separation and purification procedures. Nowadays, click chemistry reactions have become an essential means of designing and preparing separation materials; thus, interest in this synthetic technique has quickly grown. Here, the development of click chemistry and its unique advantages are briefly described firstly. The reports on click chemistry-based chromatographic separation materials published in the past five years are then systematically reviewed, focusing on two major separation fields: column chromatography and membrane chromatography. Meanwhile, recent advances in the separation materials obtained from three common types of click reactions, namely, azido-alkyne, thiol-alkene, and thiol-alkyne, are summarized. Finally, an outlook on the future of click chemistry is provided in developing efficient chromatographic separation materials.

“点击化学(click chemistry)”是由化学家Sharpless在2001年首次提出的一个合成概念^[1]^,它指的是一组强大的、几乎100%可靠的选择性反应,通过开辟以碳-杂原子键(C-X-C)合成为基础的组合化学新方法,简单高效地获得分子多样性。点击化学反应具备条件温和、反应迅速、产量高、副产物少、分离提纯简便等诸多优点,现已被广泛应用于材料科学、纳米技术、有机合成、新药研发等多个领域,Sharpless教授也因点击化学第二次获得诺贝尔化学奖。随着分离科学的不断发展,点击化学凭借其独特的优势在分离材料的构建中备受青睐。当前比较公认的点击化学反应类型包括四类,分别是环加成反应、亲核开环反应、非醇醛的羰基化学以及碳碳多键的加成反应。然而,在分离材料领域中报道最多的主要是叠氮-炔环加成、巯基-烯和巯基-炔3类点击反应(图1)。

**图1:**
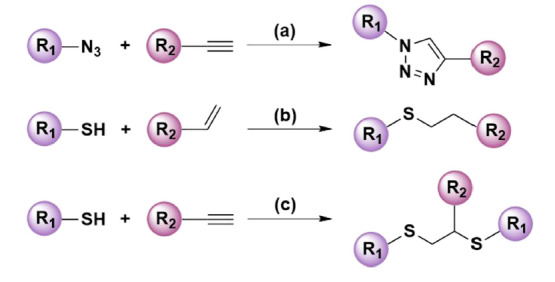
分离材料领域中常见的3种点击反应

作为最经典的点击化学反应,叠氮-炔环加成最早由Huisgen^[2]^于20世纪60年代报道,通过加热叠氮和炔的混合物可以得到两种构型的三唑基产物。21世纪初,Meldal团队^[3]^和Sharpless团队^[4]^发现,Cu(Ⅰ)的催化不仅可以加速该反应,同时能够大幅提高位置选择性,获得单一构型的三唑基产物。叠氮-炔环加成反应现已成为固定官能团的高效策略,其主要优势在于:(1)叠氮和炔官能团对大多数活性基团和广泛的溶剂、pH以及温度均具有较高的惰性;(2)叠氮化物和炔烃对水或氧的耐受性很强,可以快速引入活性官能团;(3)反应容易进行,产率高,产物中的三唑基团具有芳香性和形成氢键的能力。然而,Cu(Ⅰ)的细胞毒性在一定程度上限制了该反应在生物相关领域中的应用。另外,叠氮化合物大多具有易爆性,必须将其保存于安全、通风、阴凉的区域,在实验过程中必须严格按照操作规范进行使用,避免其在遇热、摩擦、震动、撞击或与金属接触时产生爆炸危险。

随着对点击化学研究的不断深入,巯基-烯和巯基-炔加成反应受到越来越多的关注。这类反应最初是由Posner^[5]^在1905年发现,作为无金属催化的绿色点击反应,通过自由基引发或迈克尔加成反应机理快速在烯烃键上引入巯基,生成稳定的硫醚键。该反应的主要优势在于:(1)高效,由热或光引发化学反应;(2)反应条件温和,可以在水介质中进行,适用于大范围苛刻的反应环境;(3)无需金属催化剂,无金属残留。另外,与巯基-烯点击反应相比,巯基-炔点击反应通常被认为发生了二次加成。在空间位阻允许的情况下,巯基-炔点击反应可以接枝更多的功能分子,有利于在材料表面大量修饰活性基团。

色谱分离是通过待分离物质在固定相和流动相之间分配系数的不同导致迁移速度的差异实现分离,同时利用多样性的检测手段对分离后的各组分进行分析。与其他分离策略相比,色谱分离效率高、速度快、灵敏度高,现已发展成为分析化学领域中最为重要的、应用最为广泛的分离分析方法之一。其中,色谱分离材料作为分离系统的核心组成部分,直接决定色谱的分离模式和分离效能。作为一种强大的合成工具,点击化学在色谱分离材料研究中具有广泛的应用范围和巨大的发展潜力,其设计思路通常是在固定相载体上受控引入具有叠氮基、巯基、烯基或炔基的可点击前体,然后与带有炔基、烯基、巯基和叠氮基活性基团的功能性分子进行点击接枝反应。本文重点概述了近5年点击化学在色谱分离材料研究中的最新进展,分别通过柱色谱分离和膜色谱分离两大领域展开介绍。在此基础上,探讨了点击化学在制备新型色谱分离材料过程中的关键作用和潜在优势,希望进一步为分离材料的合理设计和分离模式的精准调控提供有益参考。

## 1 基于点击化学的柱色谱分离材料

柱色谱法是将分离材料填装于柱管内作为固定相的一种色谱方法,其分离过程在色谱柱内进行,现已成为分析分离领域中适用范围最广、使用频率最高的色谱法之一。随着材料科学和色谱技术的不断发展,越来越多的研究者发现,点击化学这一类简单、高效、专一的反应可以为柱色谱分离材料的快速制备和表面修饰注入新的活力。2004年,Hoffmann团队^[6]^证明了硅胶上的叠氮基单分子层与活化的乙炔可以定量发生叠氮-炔环加成反应,提出了一种新颖的载体表面衍生化方法,这一工作推动了点击化学在色谱改性材料方面的发展。随后,Finn团队^[7]^利用叠氮-炔环加成反应将亲和配体固定在生物相容性好的琼脂糖上,制备了用于选择性亲和层析的功能化琼脂糖材料。Fréchet团队^[8]^通过叠氮-炔环加成反应分别将长链烷基和大豆胰蛋白酶抑制剂共价键合至聚合物载体上,制备了能够分离多种多肽和蛋白质的反相和亲和模式HPLC固定相。2006年,Lindner团队^[9]^将炔基化的金鸡纳生物碱衍生物共价接枝在叠氮基改性硅胶表面,制备了高效的手性固定相(CSP),证明了叠氮-炔环加成固定化策略在手性色谱分离材料构建中的实用性。同年,Liang团队^[10]^将多种炔基化的功能分子键合至叠氮基改性的二氧化硅载体上,进一步证明了点击化学是一种制备功能化HPLC固定相的有效策略。2007年,该团队再次利用点击反应开发了一系列共价键合单糖、二糖和寡糖的亲水相互作用色谱固定相,并展现了其在高效分离极性化合物方面的潜能^[11]^。此后,基于点击化学合成各式各样色谱分离材料的大门被彻底打开。现主要针对制备柱色谱分离材料过程中涉及的以下3种点击反应类型展开详细介绍。

### 1.1 基于叠氮-炔点击反应的柱色谱分离材料

由于叠氮基与炔基都比较容易被引入到所需要的化合物结构中,且生成的三唑桥环具有优异的稳定性,因此叠氮-炔环加成被认为是制备柱色谱分离材料的一种可靠和直接的方法。

一方面,叠氮-炔环加成反应可用于对环糊精手性固定相(CD-CSP)进行骨架的固定和表面功能调控^[12⇓⇓⇓-16]^。起初,Zhang等^[12]^首次以硫酸铜和抗坏血酸钠作为催化剂,将单-6-叠氮-*β*-CD(N_3_ -*β*-CD)固定在炔基化硅胶表面上,制备了新型的天然CD-CSP,成功拆分了多种手性对映异构体。同年,Wang等^[13]^以有机可溶的CuI(PPh_3_)催化叠氮-炔环加成反应制备了天然和衍生CD-CSPs,提高CD键合量的同时简化了后处理过程,极大地推动了点击化学在CD-CSP构建中的发展。最近,Li等^[15]^在多孔硅胶表面自上而下地构建了复式CD双分子层,制备了三唑桥联反向串联的天然CD-CSP。具体合成步骤如图2所示:

**图2:**
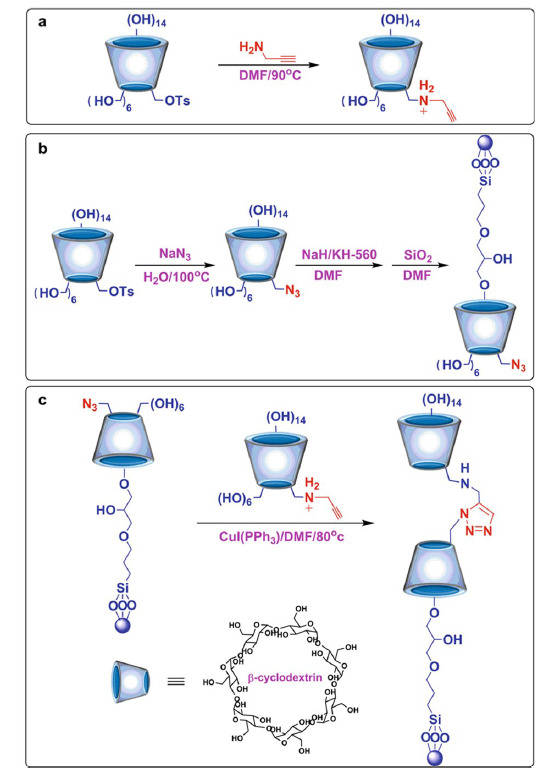
双层CD-CSP的合成途径^[15]^

首先,单-6-甲苯磺酰基-*β*-CD(TsO-*β*-CD)与丙炔胺反应,得到单-(6-脱氧-6-丙炔胺)-*β*-CD(炔基化-*β*-CD)(图2a);然后,TsO-*β*-CD与叠氮化钠反应得到N_3_-*β*-CD,再通过醚键将其固定在二氧化硅表面(图2b);最后,炔基化-*β*-CD和N_3_-*β*-CD功能化的二氧化硅之间发生点击反应(图2c),得到双层CD-CSP。CD双分子层与三唑桥链臂间的协同效应有效地提高了对丹磺酰氨基酸和小分子芳香酸的手性选择性。由此可见,叠氮-炔环加成反应不仅是设计开发CD二聚体新型功能表面材料的重要工具,同时引入的三唑桥联基团还可以提供氢键和*π*-*π*作用位点,有助于实现固定相对目标物的多重识别。除此之外,叠氮-炔环加成反应也是制备CD衍生CSP的一种重要方式,比如Jin等^[16]^将手性氨基酸L-炔丙基甘氨酸、碱性化合物丙炔胺、酸性化合物4-戊炔酸引入至醚键桥连的N_3_-CD-CSP上,对CD小口端的活性基团进行点击调节。通过键入不同的修饰基团,叠氮-炔环加成反应可以将新的手性识别位点、氢键作用以及空间位阻效应引入至天然的CD-CSP中,从而增强固定相对手性分子的识别及分离性能,扩充CD-CSP家族的同时有利于考察不同种类的衍生基团对手性分离的影响。

另一方面,除了CSP之外,叠氮-炔环加成反应也用于其他非手性柱色谱分离材料的制备^[17⇓⇓⇓-21]^,简单的一步点击反应可将功能化单体轻松引入载体中制备新型改性色谱固定相。冠醚是一类典型的大环聚醚化合物,能与金属离子、质子化胺等阳离子物质发生强相互作用。叠氮-炔环加成反应能以较高的接枝率将冠醚基团点击接枝到硅胶载体上,获得的硅基冠醚固定相表现出良好的色谱性能,不仅在反相液相色谱(RPLC)模式下能分离多环芳烃、取代苯的位置异构体和大环内酯类抗生素,同时在正相液相色谱(NPLC)模式下能有效分离C60和C70富勒烯^[17]^。糖类是天然亲水性化合物,具有多个羟基,研究一种简单有效的方法制备糖类官能化固定相具有重要意义。Wang等^[18]^采用叠氮-炔环加成点击反应制备葡萄糖和麦芽糖功能化甲基丙烯酸酯单体,然后通过单步原位共聚法制备糖类功能化整体柱,这种点击方法避免了费时且不可控的后修饰操作,优化后的整体柱对核苷类化合物、酚类化合物和苯甲酸衍生物等极性化合物具有良好的分离性能,同时可以从人类IgG胰蛋白酶消化物中高选择性且高效富集糖肽。这种单步制备糖类功能化整体柱的方法不仅为极性化合物的分离和选择性捕获提供了一种新型的亲水色谱柱,而且为整体固定相的制备提供了一种简单高效的方法。

### 1.2 基于巯基-烯点击反应的柱色谱分离材料

尽管叠氮-炔环加成反应在色谱分离材料的制备中很受欢迎,但是由于需要铜催化剂,增加了后处理的难度,越来越多的研究者们开始致力于以无金属催化的点击化学反应构建分离材料。巯基-烯加成反应具备点击化学反应的所有优点,由自由基或亲核试剂引发,无需金属催化,简化了纯化过程,具有高度的选择性,产生的副产物可忽略不计。另外,多种活化和未活化的烯类物质均可作为反应的底物,以区域选择性的方式定量生成稳定的硫醚键。于是,该反应逐渐被用于设计与制备形形色色的柱色谱分离材料。现从手性和非手性色谱分离两大应用展开介绍。

#### 1.2.1 手性色谱分离

巯基-烯点击反应可以直接将手性选择剂快速精准地键合到载体上,或者对现有手性材料进行后修饰,进一步扩充手性分离材料的种类。基于巯基-烯加成反应制备的手性柱色谱分离材料主要包括CD类^[22⇓⇓-25]^、奎宁(QN)类^[26⇓⇓⇓⇓-31]^、纤维素类^[32,33]^、氨基酸类^[34⇓⇓-37]^以及其他手性选择剂修饰的CSPs^[38⇓⇓-41]^。

CD的空腔结构及手性中心使其能够与大量手性分子形成主客体包合物,因此,被广泛用作手性色谱分离中的手性选择剂。通过巯基-烯点击反应,将单巯基取代的*β*-CD共价接枝在双键功能化的硅胶载体上,可以合成结构明确可控的单(6-巯基-6-去氧)-*β*-CD-CSP(图3a),最大限度地保留天然CD的本征结构^[24]^。由于桥联臂无识别作用位点,这种键合方式非常有利于对天然CD的本征手性识别能力进行考察。通过与三唑桥联CD-CSP和咪唑嗡桥联CD-CSP进行手性拆分行为比较发现,功能性桥联臂在提升部分对映体选择性的同时也会小幅损失CD的本征手性识别能力。因此,采取不同类型的点击化学反应可以引入不同功能的桥联臂,为CD-CSP结构的精准设计以及手性样品的高效拆分提供有益参考。

**图3:**
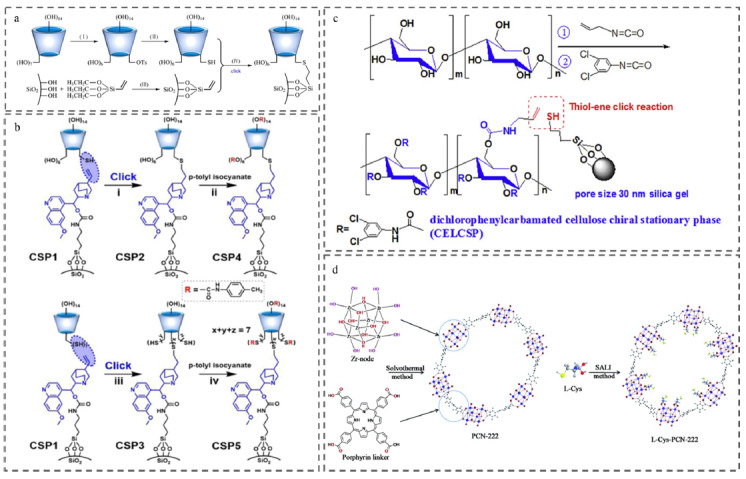
基于巯基-烯反应制备的手性色谱分离材料

为了发展高度通用的色谱分离材料体系,Lu等^[31]^通过简单的巯基-烯表面点击反应制备了一系列结构明确的QN桥联CD复式手性结构(图3b)。其中QN和CD不仅能很好地展示出各自的分离能力,实现分离通量的互补,而且对各自难以分离的分析物展现出协同增强的作用,在保持高选择性的同时成倍提升了分离通量。所构建的双相CSP对多种液相色谱模式具有良好的适用性和稳定性。简单高效的巯基-烯点击反应将QN和CD有效键合,推动了这种相对通用的双相手性拆分平台的进一步发展。

纤维素及其衍生物也是较为通用的手性选择剂。Li等^[32]^将烯丙基纤维素的衍生物通过热引发剂点击接枝在巯基丙基硅胶上,制备了纤维素键合固定相(图3c),可以在RPLC模式下成功地用于6种手性杀菌剂对映体的拆分。巯基-烯加成反应的温和条件有利于维持纤维素的有序立体结构,同时定向合成的高产率也能提供足够的手性基团键合量,使新固定相具有良好的手性色谱性能。

L-半胱氨酸(L-Cys)作为一种常见的手性选择剂,其结构本身含有的巯基可以直接用于点击反应。例如,Gao等^[37]^将一种新型的L-Cys修饰的手性锆基金属有机骨架(L-Cys-PCN-222)点击连接到带有烯基的毛细管壁上(图3d),成功制备了键合型开管柱,有效分离了17种手性化合物,包括氨基酸、咪唑啉酮、芳酮羧酸类农药以及氟喹诺酮类化合物。

#### 1.2.2 非手性色谱分离

巯基-烯点击反应为非手性色谱分离材料的合成和化学修饰开辟了新的途径,通过将一种或多种类型的功能分子引入色谱载体中,实现了色谱材料和分离模式的多样化。这些功能分子主要包括疏水或亲水基团^[42⇓⇓⇓-46]^、离子液体^[47⇓⇓⇓⇓⇓-53]^、两性离子^[54,55]^、交联共聚物^[56⇓⇓-59]^及其他功能单体^[60,61]^等。

通过引入疏水或亲水基团对固定相进行亲疏水改性,可以有针对性地高效分离目标物。张丽华课题组^[42]^提出了一种新型功能化的多孔整体材料制备策略(图4a),通过一步溶胶-凝胶反应制备了含有大量乙烯基的大孔/介孔有机硅整体材料,丰富的双键分布为巯基-烯点击化学反应修饰高密度官能团提供了大量活性位点,该方法获得了C18基团在杂化整体材料上的高表面覆盖率,进一步证明了其在烷基苯和多肽的RPLC模式中的适用性。

**图4:**
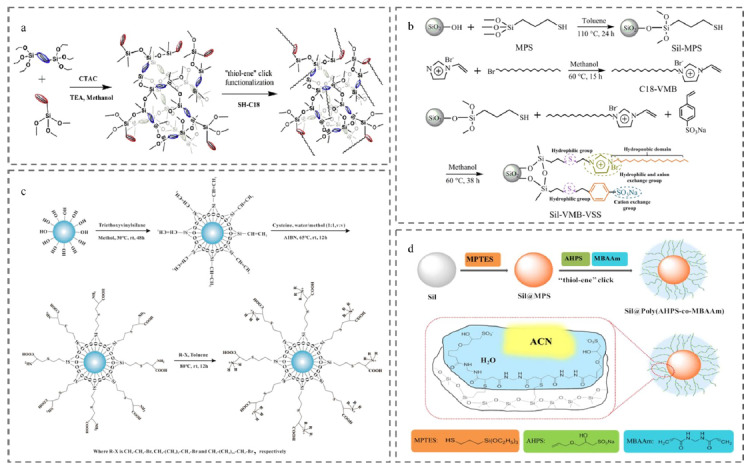
基于巯基-烯反应制备的非手性色谱分离材料

离子液体指的是一类在室温或接近室温条件下呈液态、完全由有机阳离子和有机或无机阴离子组成的液体。得益于其优异的理化性质,离子液体逐渐发展成为色谱分离的理想材料。通过对离子液体进行功能化设计,从而引入不同的功能性基团,可以实现多种分离需求。Ren等^[48]^采用硫醇-烯点击反应,将嵌有咪唑基离子液体的C18链和芳基磺酸盐两种单体成功修饰到二氧化硅的表面(Sil-VMB-VSS),制备了一种新型的反相/亲水/离子交换混合模式液相色谱固定相(图4b),其多模式保留能力可以为各种类型的化合物和复杂样品提供灵活的选择性。高效的巯基-烯点击反应使多种功能基团同时修饰载体成为可能,为合成混合模式固定相提供了一种可行的途径。

氨基酸作为典型的两性离子化合物,常被用作两性离子功能分子制备固定相。Wang等^[55]^基于Cys与乙烯基功能化二氧化硅之间的巯基-烯点击化学反应,制备了Cys两性离子固定相。另外,分别用溴乙烷、1-溴辛烷和1-溴十八烷进行改性,得到了一系列季铵功能化的固定相(图4c),实现了在反相/离子交换混合模式下对酸性和碱性蛋白质的选择性分离。这种简单的点击反应推动了两性离子固定相在蛋白质分离方面的进一步应用。

聚合物刷因具有三维纳米结构的柔性聚合物链,可以提供丰富的功能结合位点,增加材料和目标之间的相互作用。Shao等^[56]^把巯基-烯表面点击聚合作为合成聚合物刷的有力工具,以*N*,*N*'-亚甲基双丙烯酰胺(MBAAm)为交联剂,3-烯丙氧基-2-羟基-1-丙磺酸钠盐(AHPS)为单体,将支链共聚物接枝至巯基二氧化硅上(图4d),制备出亲水/阳离子交换固定相Sil@Poly (AHPS-co-MBAAm)。最后基于混合模式保留机制,成功实现了核苷、碱基和酸性化合物的高效分离以及糖肽的高选择性富集。这种由巯基-烯点击反应接枝的三维支链共聚物提供了丰富的功能结合位点,赋予了二氧化硅颗粒较高的亲水性和协同的静电作用,进一步提升了固定相对目标物的选择性和分离效率。

### 1.3 基于巯基-炔点击反应的柱色谱分离材料

巯基-炔点击反应通常被认为是巯基与三键、双键之间的次序加成反应,是巯基-烯加成反应的重要延伸与拓展。该点击反应类型最早由Bowman课题组^[62]^在2009年提出,目标是在巯基-烯点击反应的基础之上,继续发展一系列可靠且高选择性的碳-硫成键反应来实现原子之间的高效连接,有利于新型高性能材料的模块化制备。目前,巯基-炔反应作为一种强有力的合成手段,已逐渐渗透进入色谱分离领域,在构建交联型^[63⇓-65]^、聚合型材料^[66]^以及手性修饰^[67]^等研究中发挥重要作用。

Shields等^[63]^通过将1,4-二乙炔基苯和1,6-己二硫醇引入到巯基功能化的二氧化硅表面,制备了在极端pH条件下非常稳定的高交联固定相,结果表明,使用巯基-炔反应有望创制稳定和多样化的固定相,通过添加不同的共聚物和单体,有可能产生多种类型的新相。Ma等^[66]^采用光引发的巯基-炔聚合反应,通过与多种类型的硫醇共聚,在紫外透明的熔融二氧化硅毛细管内原位制备多种杂化单体。这些杂化单体产生了具有较大贯通孔隙尺寸/骨架尺寸比的双连续多孔结构,使得毛细管整体柱对复杂样品具有较强的分离能力,成功应用于牛血清白蛋白胰酶消化物的分离。这种光引发的巯基-炔聚合可在10 min内完成,比溶胶-凝胶化学、热引发自由基聚合和开环聚合等合成杂化整体柱的方式更加节省时间,为其他杂化整体材料的制备提供了一种简便快速的方式。另外,严秀平团队^[67]^利用巯基-炔点击反应将3种具有不同手性中心的结构,包括1-巯基甘油(TGC)、巯基琥珀酸(MSA)和*N*-乙酰半胱氨酸(NAC)共价连接在微孔有机网络(MONs)材料上(图5),成功制备了毛细管柱并实现了多种手性化合物的气相色谱拆分,其分辨率和选择性优于3种商用手性毛细管柱。这种基于巯基-炔点击反应的手性后修饰策略,为合成手性MONs材料提供了一种简单可行的方法,极大地开发了手性MONs材料应用在手性分离方面的巨大潜力。

**图5:**
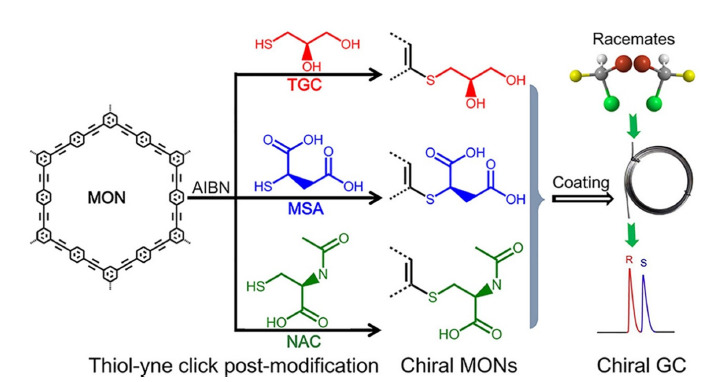
巯基-炔点击策略用于手性气相色谱的后合成手性MONs的示意图^[67]^

## 2 基于点击化学的膜色谱分离材料

膜分离技术是源于20世纪初期并在20世纪60年代迅速崛起的一门新兴的分离技术。膜分离技术以具有选择性分离功能的分离膜为核心,借助外界力量或以化学位差为推动力,实现不同组分的分离、纯化与浓缩。膜分离通常在常温环境下进行、无相态变化或化学变化、简单易操控、节能环保、可在分子级别内进行物质分离,现已被广泛应用于食品、医药、水处理等多个研究领域中,逐渐发展成为分离科学中强有力的分离手段之一。其中,膜色谱是将膜分离技术和色谱法有机地结合起来,采用一定孔径的膜作为介质,在其表面或孔隙中键合功能分子,利用目标分子与膜的相互作用进行分离纯化。膜色谱融合了膜分离技术样品容量大、压力低和色谱法选择性高、分析速度快的优点,逐渐发展成为色谱分离领域中的一个重要分支。尽管目前很多膜材料并未被真正地应用于膜色谱分离技术中,但其通过对膜基质进行功能改性的设计思路与膜色谱原理基本一致。因此,本文将这些具有膜色谱分离潜能的膜材料统称为膜色谱分离材料。作为整个分离系统中最为关键的组成部分,其结构的设计与优化是十分重要的。起初,Cai等^[68]^采用叠氮-炔环加成反应成功制备了聚偏氟乙烯接枝共聚物膜,这一工作极大地刺激了点击化学在膜领域的发展。与传统合成策略相比,点击化学凭借其反应条件温和、反应速率快、选择性高等优点为膜的合成与表面改性提供了更精确、可控和高效的平台。现针对3种常见的点击反应类型在膜色谱分离材料制备中的应用加以介绍和讨论。

### 2.1 基于叠氮-炔点击反应的膜色谱分离材料

利用具有高选择的叠氮-炔环加成反应可以在膜表面锚定多种类型的功能分子,实现膜表面官能化。通常是先将膜表面或成膜聚合物进行叠氮或炔基功能化,再通过简单的一步点击反应,将炔基或叠氮官能化分子接枝到膜上。不同的功能分子赋予膜材料多样性的功能与应用。基于叠氮-炔环加成反应构建的膜色谱分离材料主要在提高膜防污性能^[69⇓⇓-72]^和物质选择性分离^[73⇓-75]^领域中受到了广泛应用。

膜污染一直是限制其快速发展的瓶颈问题。膜的实际应用条件和分离系统非常复杂,常常伴随多种类型污染物的存在,容易造成膜结构的不良变化和分离性能的显著恶化,大大缩短膜的使用寿命。为了克服膜污染的问题,制备高效的防污膜被视为从源头上提高防污性能最有效、最基本的方法。林立刚团队^[70]^对乙烯-乙烯醇共聚物膜进行炔基功能化,然后利用点击反应将叠氮化的*β*-CDs固定在其表面。经过处理后,亲水膜的油水分离性能显著提高,且*β*-CDs的“分子笼”几何结构可以诱导多种客体分子进入空腔,对可溶性有机污染物表现出良好的吸附性能。最终,通过简单的一步膜过滤操作即可阻断乳化油滴,处理混合污染物废水。除此之外,该研究组还提出了一种基于*β*-CDs的滑动超分子聚合物刷(SSPBs)动态表面修饰的膜制备方法^[71]^(图6)。通过叠氮-炔环加成反应,将具有三维亲水性结构的SSPBs引入到乙烯-乙烯醇共聚物膜基质中。滑动的亲水环提供了一个主动排斥系统,提高水通量的同时减少了多种污染物与膜表面直接接触。这种三维膜表现出较高的表面亲水性、水下疏油性和防污性。可以看出,基于叠氮-炔环加成反应的三维亲水膜制备策略简单高效,为膜的功能化修饰提供了思路。

**图6:**
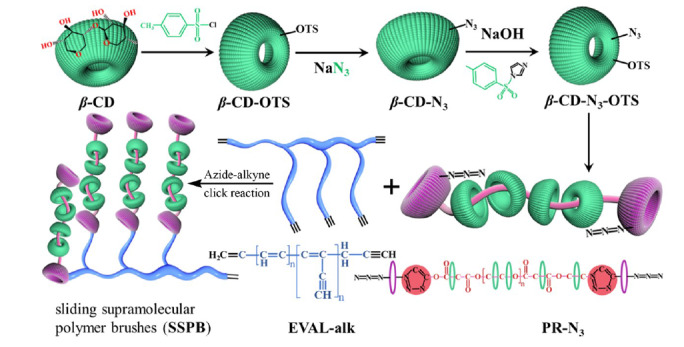
通过叠氮-炔点击耦合反应形成滑动超分子聚合物刷的策略示意图^[71]^

另外,对分离膜进行合理设计有助于实现对特定物质的高亲和性和高通量筛选。Tatsuo Maruyama团队^[73]^尝试了一种基于点击化学的相分离制备亲和膜的新方法。在含叠氮的表面活性剂存在下,通过相分离法制备了多孔聚合物膜,然后再与水中的炔基化分子点击结合,完成在膜表面上的固定化。结果显示,两种不同的炔基化分子修饰的聚合物膜分别对六聚组氨酸标记蛋白和万古霉素展现出较强的亲和性,使其在复杂基质中对两种物质的分离纯化成为可能。由于点击固定化是在水中进行的,因此这种点击反应进一步拓宽了多种水溶性功能分子在制备亲和膜中的应用范围。

### 2.2 基于巯基-烯点击反应的膜色谱分离材料

近年来,采用巯基-烯点击化学反应快速制备或功能化膜分离材料的报道越来越多,获得的分离膜因其独特的结构和性能在油水分离^[76⇓⇓⇓-80]^、气体分离^[81⇓⇓-84]^、水环境污染物去除^[85⇓⇓⇓-89]^以及其他物质的选择性分离领域^[90⇓-92]^中有着广阔的应用前景。

日常生活、工业生产以及石油泄漏等造成的油污染日益严重,现已成为全球性的环境挑战之一。如何快速有效地处理含油废水已成为水净化领域亟待解决的问题。得益于膜独特的分离性能和可调节的孔隙率,膜分离已被公认为是一种高效的油水分离方法。然而,油在膜表面的吸附和聚集容易造成膜污染,严重影响膜的分离效率和使用寿命,因此,提高膜的防污性能同样至关重要。Shen等^[77]^提出了一种巯基-烯点击聚合方法用于制备具有超亲水和超疏油能力的聚乙二醇(PEG)修饰的聚丙烯腈(PAN)膜(图7a)。首先用碱溶液将原始的PAN膜水解,然后与半胱胺盐酸盐反应生成巯基化膜表面,最后将聚乙二醇甲醚甲基丙烯酸酯(PEGMA)通过点击反应共价接枝到膜表面,得到PEG修饰的PAN膜。该膜对几种水包油乳化剂的分离效率高达99.9%,展现出良好的油水分离性能。研究还发现,即使经过3次循环过滤,该膜的分离通量也能恢复到95%以上的高水平,表明其在分离过程中具有较强的抗污能力。由此可见,巯基-烯点击反应可以作为一种巧妙的接枝途径,用于油水分离膜表面结构的设计与改性。

**图7:**
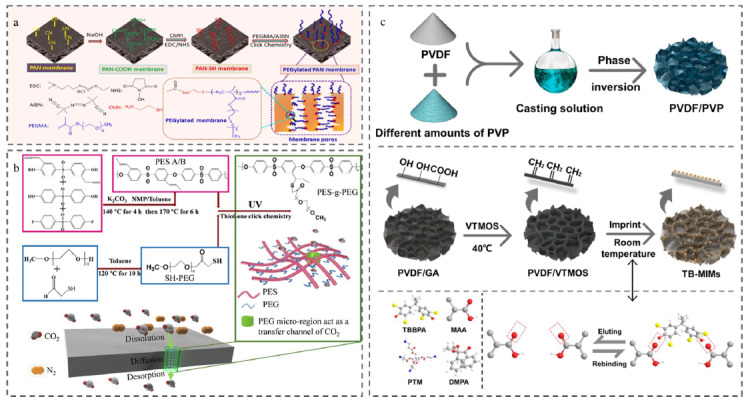
基于巯基-烯点击反应制备的膜分离材料

除油污染以外,CO_2_的大量排放也严重影响地球的生态环境,发展高效、可持续的CO_2_分离方法对碳捕获和再利用技术至关重要。膜分离作为一种绿色分离技术,因其分离效率高、能耗低、稳定性好而被认为是分离CO_2_的理想策略。利用巯基-烯点击化学方法,可制备一种以聚醚砜(PES)为主链提供机械支撑、低相对分子质量的PEG为侧链提供CO_2_运输通道的梳状共聚物膜(图7b)^[82]^。通过改变PEG侧链的长度和密度可调控微相分离的精细结构,进而实现共聚物膜对CO_2_的最佳分离性能,优化后的共聚物膜对CO_2_显示出优异的渗透性和选择性。可以看出,基于简单的巯基-烯点击化学反应对共聚物材料的微观结构进行调控有助于进一步优化膜的气体分离性能。

另外,人类活动排放的大量污染物进入水体会使水环境的理化性质和生物组成发生改变,进而造成水质恶化,破坏生态环境,甚至危害人体健康。目前,利用经济环保的膜分离技术去除水环境中的污染物备受关注。其中,具有分子选择性的分子印迹膜材料可在膜及其孔表面形成选择性的再结合位点,在特异性识别和去除污染物方面表现出前所未有的优越性。Yu等^[85]^利用巯基-烯反应制备了一种对四溴双酚A具有精确捕获和分离能力的分子印迹膜材料(图7c),在不同化学环境中均表现出显著的稳定性,为选择性去除水环境中的四溴双酚A提供了可能。巯基-烯这种特异性点击反应为定制分子印迹膜材料提供了新的思路,进一步推动高选择性识别和分离技术的发展。

### 2.3 基于巯基-炔点击反应的膜色谱分离材料

巯基-炔点击反应已被报道用于各种用途的功能材料的合成,特别在聚合物的制备中,该方法有望形成均匀稳定的网络结构。在膜分离材料领域中,常采用巯基-炔点击反应制备聚合物膜用于分离环境中的污染物^[93⇓-95]^和气体^[96]^等。

纳米复合膜是将连续的致密相和分散的多孔相结合,利用聚合物膜作为结构支撑,多孔纳米材料作为快速传输通道。其中,实现无填料堆积和无缺陷是赋予纳米复合膜高渗透性和高选择性的关键挑战。Chen等^[93]^引入了一种界面桥接策略,将含炔基的共价有机骨架(COF)通过巯基-炔点击反应初步接枝到亲水性Cys桥上,然后再与聚乙烯亚胺(PEI)和均苯三甲酰氯(TMC)交联,最终得到聚酰胺复合膜(PA/COF-C膜)(图8)。这种基于点击化学的亲水性改进策略最大限度地提高了COF材料与聚合物膜的相容性并减少了膜缺陷。优化后的纳米复合膜材料对多种染料和抗生素具有优异的渗透性和选择性,展现出在工业废水处理方面的应用潜力。

**图8:**
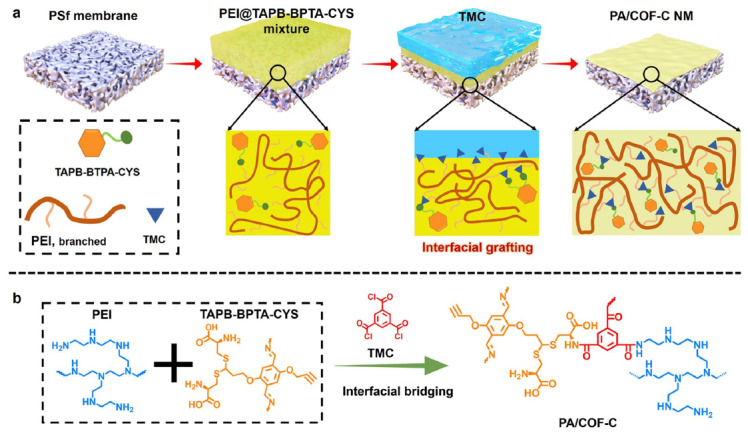
(a)通过界面桥接方法制备PA/COF-C纳米复合膜示意图和(b)PA/COF-C层的合成化学反应^[93]^

聚合物独特的理化性质决定了其在气体传输方面应用的可能,适当地改变网络结构可以调控它们的分离性能。高交联的蓖麻油基聚氨酯膜因通过巯基-炔点击反应而形成了具有良好结构的交联网络和灵活的柔性硫醚键,展现出优异的力学性能和热稳定性,为气体高效输送提供保障^[96]^。结构的优化使得该膜对CO_2_具有较高的选择性,可以高效分离CH_4_和H_2_中的CO_2_。

## 3 总结与展望

本文综述了近5年来点击化学在色谱分离材料研究方面的最新进展,主要从柱色谱分离和膜色谱分离两个方面对叠氮-炔环加成、巯基-烯和巯基-炔加成3种常用的点击反应类型进行了讨论。可以看出,尽管点击化学的发展历时较短,但其凭借独特的优势在分离科学领域得到了迅猛发展,尤其是基于巯基加成反应的应用显示出日益增长的趋势。点击化学不仅为色谱分离材料的快速合成与精准修饰提供了一种强大高效的工具,同时有助于进一步提升材料的分离性能,例如叠氮-炔环加成反应生成的三唑杂环能够提供氢键、*π*-*π*作用位点或静电作用位点,增强材料和目标物之间的相互作用;巯基-炔反应经首次加成后生成的双键可为其他功能分子提供额外的二次加成接枝位点,有利于实现分离功能互补;简单的点击反应操作扩大了单体的选择范围,丰富了表面功能多样化整体柱的制备工艺,且较高的产率显著提高了整体柱的柱效等。相信未来仍有大量的相关工作值得去探索,主要挑战集中在设计更新颖的分离材料、开发更多专属性的反应类型、提高功能分子的键合效率以及改善材料的分离效能等方面,以继续深度挖掘点击化学的潜在应用价值。
